# CREATING STRUCTURES FOR SUSTAINABILITY OF EVIDENCE-BASED DEMENTIA CAREGIVER PROGRAMS

**DOI:** 10.1093/geroni/igad104.1015

**Published:** 2023-12-21

**Authors:** Carey Wexler Sherman, Joseph Gaugler

## Abstract

Ensuring sustainable impact for broad-based evidence-based programs (EBPs) serving caregivers for persons living with dementia in the U.S. requires systemic maintenance of programmatic fidelity. This symposium aims to assist decision makers, aging and public health networks, and intervention developers to anticipate and deploy effective strategies and resources required to implement EBP interventions and sustain them. A series of four papers describes precursor studies and activities leading to the development of a structure aimed at ensuring that one such EBP, the Savvy Caregiver® (Savvy), can be faithfully sustained by sponsoring organizations. The first paper presents results from in-depth qualitative interviews of key staff (n=25) in nine organizations delivering Savvy. Findings highlight organizational, structural, and regional collaborations that contributed to sustained EBP implementation with fidelity. The second paper describes an online program to train Savvy interventionists. Informed by the first paper’s findings a fully asynchronous multi-media professional continuing educational interventionist training program was developed in collaboration with the educational design team at Emory’s School of Nursing. The third paper presents results of the online trainer program evaluation conducted among 76 interventionists from 32 organizations in eight states. High scores on content comprehension (95-99%) and participant feedback document benefits of self-paced learning, interactive materials and quizzes, and self-reflective exercises. The final paper describes a platform (Savvy Systems, LLC) created to ensure Savvy’s long-term dissemination, programmatic integrity and sustained use. The single platform provides for authorized content dissemination, leader training, enhanced referrals, and copyright compliance through licensing. Discussant will offer policy and practice implications. This is a collaborative symposium between the Alzheimer’s Disease and Related Dementias and Family Caregiving Interest Groups.

